# Xylan degradation by the human gut *Bacteroides xylanisolvens* XB1A^T^ involves two distinct gene clusters that are linked at the transcriptional level

**DOI:** 10.1186/s12864-016-2680-8

**Published:** 2016-05-04

**Authors:** Jordane Despres, Evelyne Forano, Pascale Lepercq, Sophie Comtet-Marre, Gregory Jubelin, Christophe Chambon, Carl J. Yeoman, Margaret E. Berg Miller, Christopher J. Fields, Eric Martens, Nicolas Terrapon, Bernard Henrissat, Bryan A. White, Pascale Mosoni

**Affiliations:** Institut National de la recherche Agronomique (INRA), UR454 Microbiologie, Centre de Clermont-Ferrand-Theix, 63122 Saint-Genès-Champanelle, France; INRA, Plate-forme d’Exploration du Métabolisme, 63122 Saint-Genès Champanelle, France; Department of Animal and Range Sciences, Montana State University, Bozeman, MT 59718 USA; Department of Animal Sciences, University of Illinois at Urbana-Champaign, Urbana, IL USA; Institute for Genomic Biology, University of Illinois at Urbana-Champaign, Urbana, IL USA; Department of Microbiology and Immunology, University of Michigan Medical School, Ann Arbor, MI 48109 USA; Architecture et Fonction des Macromolécules Biologiques (AFMB), UMR 7257 CNRS, Université Aix-Marseille, 163 Avenue de Luminy, 13288 Marseille, France; INRA, USC 1408 AFMB, 13288 Marseille, France; Department of Biological Sciences, King Abdulaziz University, Jeddah, Saudi Arabia

**Keywords:** Xylan degradation, Human gut, *Bacteroides*, Polysaccharide-Utilization Locus, CAZymes, RNA-seq, Proteomics, Mutagenesis

## Abstract

**Background:**

Plant cell wall (PCW) polysaccharides and especially xylans constitute an important part of human diet. Xylans are not degraded by human digestive enzymes in the upper digestive tract and therefore reach the colon where they are subjected to extensive degradation by some members of the symbiotic microbiota. Xylanolytic bacteria are the first degraders of these complex polysaccharides and they release breakdown products that can have beneficial effects on human health. In order to understand better how these bacteria metabolize xylans in the colon, this study was undertaken to investigate xylan breakdown by the prominent human gut symbiont *Bacteroides xylanisolvens* XB1A^T^.

**Results:**

Transcriptomic analyses of *B. xylanisolvens* XB1A^T^ grown on insoluble oat-spelt xylan (OSX) at mid- and late-log phases highlighted genes in a polysaccharide utilization locus (PUL), hereafter called PUL 43, and genes in a fragmentary remnant of another PUL, hereafter referred to as rPUL 70, which were highly overexpressed on OSX relative to glucose. Proteomic analyses supported the up-regulation of several genes belonging to PUL 43 and showed the important over-production of a CBM4-containing GH10 endo-xylanase. We also show that PUL 43 is organized in two operons and that the knockout of the PUL 43 sensor/regulator HTCS gene blocked the growth of the mutant on insoluble OSX and soluble wheat arabinoxylan (WAX). The mutation not only repressed gene expression in the PUL 43 operons but also repressed gene expression in rPUL 70.

**Conclusion:**

This study shows that xylan degradation by *B. xylanisolvens* XB1A^T^ is orchestrated by one PUL and one PUL remnant that are linked at the transcriptional level. Coupled to studies on other xylanolytic *Bacteroides* species, our data emphasize the importance of one peculiar CBM4-containing GH10 endo-xylanase in xylan breakdown and that this modular enzyme may be used as a functional marker of xylan degradation in the human gut. Our results also suggest that *B. xylanisolvens* XB1A^T^ has specialized in the degradation of xylans of low complexity. This functional feature may provide a niche to all xylanolytic bacteria harboring similar PULs. Further functional and ecological studies on fibrolytic *Bacteroides* species are needed to better understand their role in dietary fiber degradation and their impact on intestinal health.

**Electronic supplementary material:**

The online version of this article (doi:10.1186/s12864-016-2680-8) contains supplementary material, which is available to authorized users.

## Background

Plant cell wall (PCW) polysaccharides *i.e. *cellulose, hemicelluloses and pectins present in cereals, fruits and vegetables are an important part of the dietary fibers consumed by humans. These polysaccharides are not digested in the upper digestive tract and reach the colon where they are degraded and fermented by the symbiotic intestinal microbiota. It is estimated that the amount of PCW polysaccharides that reach the colon every day is between 10 and 25 g [1], hemicelluloses representing up to 30 % of plant dry weight [2, 3].

Two hemicellulosic polysaccharides that are widely consumed by humans consist primarily of glucuronoarabinoxylans (found in grasses and cereals) and arabinoxylans (mainly found in cereal grains), and both belong to the xylan family of plant polysaccharides [2]. Structurally, xylans consist mostly of linear backbone of β-1,4-D-xylopyranoside units which are commonly decorated with variable numbers 4-O-methyl-glucuronyl, acetyl, feruloyl, and arabinofuranosyl substituents. Depolymerization of xylans therefore requires the complementary action of enzymes acting on the Xyl backbone *i.e.* endo-β-1,4-xylanase, β-xylosidase, and of enzymes debranching the substituents *i.e.* α-L-arabinofuranosidase, α-glucuronidase, acetyl- and feruloyl-xylan esterases [2, 4]. In general, the enzymatic degradation of xylans leads to mixtures of oligosaccharides of different degree of polymerization and of various linkage compositions [5].

Xylans and some of their breakdown products *i.e*. xylo-oligosaccharides (XOS) and arabinoxylan-oligosaccharides (AXOS) have been shown to have a prebiotic effect and to provide health benefits, particularly in the management of obesity and related disorders [6, 7]. These health benefits are strongly correlated with changes in the composition of the gut microbiota: increase in *Bifidobacteria/Lactobacilli* as well as in *Bacteroides/Prevotella* and *Roseburia* groups. Members of the *Bacteroides, Prevotella* and *Roseburia* genera from the human colon (or from the rumen) have been described for their xylanolytic activity [8–13]. These species can produce XOS or AXOS of low degree of polymerization that can be utilized by glycolytic bacteria like *Bifidobacteria* [6, 13, 14]. Hence the primary degraders of xylans play an important role in generating prebiotics in situ from PCW polysaccharides. That is why it is essential to fully understand the xylanolytic enzyme systems of human gut bacteria, in order to identify which bacterial species play a key role in generating oligosaccharides from PCW polysaccharides that are beneficial to the host.

In a recent article, Rogowski et al. [13] highlighted the complexity of the xylanolytic enzyme system of *Bacteroides ovatus* ATCC 8483^T^*.* Here we present a combination of transcriptomics, proteomics and mutagenesis approaches to understand the xylanolytic function of another prevalent human gut symbiont, *Bacteroides xylanisolvens* XB1A^T^ [15], for which we previously showed that it displays a high xylan-degrading activity [9, 10, 16]. Indeed, the genome of this species harbors 256 genes encoding degradative carbohydrate-active enzymes (CAZymes, http://www.cazy.org/) including glycoside hydrolases (GH) and carbohydrate esterases (CE) potentially active on xylans (GH10, GH51, GH67, GH115, CE1, CE6). In addition, most of these genes were found to be clustered in genome loci called polysaccharide utilization loci (PUL). PUL function has been extensively dissected in *Bacteroides thetaiotaomicron* VPI-5482 (see reviews [17, 18]) but needs to be better decrypted in other species of the *Bacteroides/Prevotella* genus involved in PCW degradation like *B. xylanisolvens. B. xylanisolvens* XB1A^T^ genome was predicted to contain 74 PULs by Terrapon et al. [19], and the PUL numbering used hereafter is that given in the PULDB database: http://www.cazy.org/PULDB/.

## Results

### Transcriptomic and proteomic analyses of *B. xylanisolvens* XB1A^T^ grown on insoluble oat-spelt xylan (OSX)

#### Transcriptomic analysis

RNA-seq data (Illumina Hiseq 2000) were obtained from *B. xylanisolvens* XB1A^T^ grown on insoluble oat-spelt xylan (OSX) at mid- and late-log phase relative to glucose and/or xylose (Table 1) (Additional file 1: Figure S1).

**Table 1:** Analyses performed from *B. xylanisolvens* XB1A^T^ cultures according to substrate and growth phase^a^

	Mid-log phase		Late-log phase		
Glucose	RNA-seq	RT-qPCR	RNA-seq	nd	nd
Xylose	nd	nd	RNA-seq	RT-qPCR	Proteomics
Oat-spelt xylan	RNA-seq	RT-qPCR	RNA-seq^b^	RT-qPCR	Proteomics

^a^Not done

^b^Data that were discarded (see Additional file 1: Table S1)

**Fig. 1:**
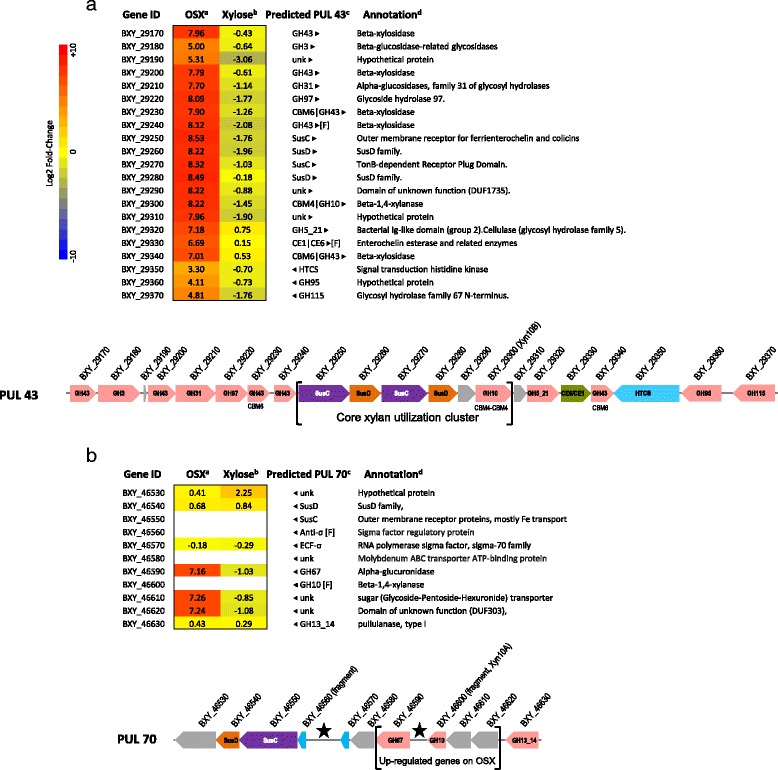
Polysaccharide Utilization Loci up- or down-regulated on insoluble oat-spelt xylan (OSX) or xylose, compared with glucose. Heatmap and gene organization of PUL 43 (**a**) and PUL 70 (**b**). ^a^Heatmap based on Log2 Fold-Change of gene expression on OSX relative to glucose with *B. xylanisolvens* XB1A^T^ harvested at mid-log phase. ^b^Heatmap based on Log2 Fold-Change of gene expression on xylose relative to glucose with *B. xylanisolvens* XB1A^T^ harvested at late-Log phase. ^c^PUL predicted in *Bacteroides xylanisolvens* XB1A^T^ (http://www.cazy.org/PULDB/) and presented below the heatmap. The color code used for carbohydrate-active enzymes highlights the nature of the main functional module: glycoside hydrolase (*light pink*) or carbohydrate esterase (*light brown*). PUL marker genes, *susC*- and *susD*-like genes, are represented by purple and orange boxes, respectively, whilst the regulator genes appear in cyan. Other genes predicted as members of the PULs are shown in grey. Genomic regions containing N stretches and/or unassigned genes are marked with a star. ^d^Automatic annotation of *Bacteroides xylanisolvens* XB1A^T^ genome available at http://www.ncbi.nlm.nih.gov/genome/?term=xylanisolvens. No Log2 Fold-change values were obtained (*white boxes*) for ORFs or fragments of ORFs [F] not detected by automatic annotation because of sequence gaps (N stretches) in the genomic region of interest; these ORFs were manually annotated in this study

Transcriptional analysis of *B. xylanisolvens* XB1A^T^ grown on OSX at mid-log phase revealed the strong expression (Log2 Fold-Change > 5 compared to growth on glucose – Fig. 1) of several genes localized within two PULs, *i.e*. PUL 43 (BXY_29170 to BXY_29370) and PUL 70 (BXY_46530 to BXY_46630). Besides these two PULs, 302 genes were also up-regulated (2 ≤ Log2FC < 5) on OSX relative to glucose and approximately half of these genes belonged to predicted PULs or to gene clusters putatively involved in carbohydrate and energy metabolism. Interestingly, within these moderately induced PULs, 15 PULs (*i.e*. PUL 5, PUL 10, PUL 14, PUL 22, PUL 29, PUL 34, PUL 37, PUL 39, PUL 42, PUL 46, PUL 58, PUL 65, PUL 71, PUL 73, PUL 74) were also induced on citrus pectin [20], whereas PUL 43 and PUL 70 were specifically induced on OSX (Additional file 1: Figure S2).

PUL 43 is a cluster of 21 genes, including 13 genes with annotations consistent with xylan degradation, *i.e.* 12 glycoside hydrolases of families GH3, GH5, GH10, GH43, GH95 and GH115 and a bimodular carbohydrate esterase of families CE1 and CE6, in addition to two tandemly arranged *susC/D*-like genes encoding extracellular membrane proteins potentially involved in the sequestration/internalization of xylan or resulting oligosaccharides into the cell, one gene encoding a hybrid two component system (HTCS) and two genes of unknown function (Fig. 1a).

PUL 70 is predicted in a genomic locus containing sequence gaps (poly-N stretches) and therefore was not fully annotated; however partial gene sequences allowed us to improve manually the annotation of this PUL (Fig. 1b). PUL 70 contains 11 genes encoding four GH proteins of families GH2, GH10, GH13 and GH67, SusC/D-like proteins, two putative sugar transporters, two regulatory proteins (anti-σ factor/ECF-σ) and one unknown protein.

**Fig. 2:**
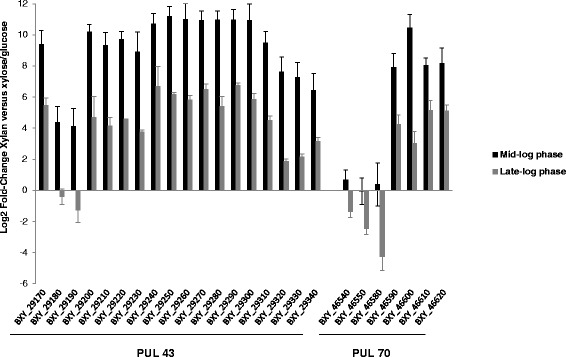
Impact of growth substrate on gene expression in PUL 43 and rPUL 70. Relative expression measured by RT-qPCR of selected genes from PUL 43 and rPUL 70 on OSX versus glucose at mid-log phase or OSX versus xylose at late-log phase. Differences in gene expression between mid- and late-log phase were significant for all targeted genes (*P* < 0.01)

When comparing the RNA-seq heatmaps of the two PULs, one can notice that all the genes of PUL 43 were up-regulated upon growth on OSX, whereas only the expression of three genes of PUL 70 were highly induced namely genes encoding two glycoside hydrolases (GH2, GH67) and one sugar transporter (Fig. 1b). In order to confirm these RNA-seq data and to get information on the expression of important genes in PUL 70 (BXY_46650, BXY_46580, and BXY_46600), relative RT-qPCR was performed on a subset of PUL 43 and PUL 70 genes (Fig. 2). The results confirmed the over-expression of PUL 43 genes upon growth on OSX relative to glucose at mid-log phase. In PUL 70, we could verify the absence of induction of BXY_46540 and BXY_46550 encoding SusC- and SusD-like proteins as well as of BXY_46580 encoding a putative ABC transporter, and the over-expression of BXY_46600 encoding a GH10 endo-xylanase. Relative RT-qPCR was also carried out with *B. xylanisolvens* XB1A^T^ grown on OSX at late-log phase. As expected, gene over-expression was higher at mid-log phase than at late-log phase, except for two genes (BXY_29180, BXY_29190) in PUL 43 and three genes in PUL 70 (BXY_46540, BXY_46550, BXY_46580) that were down-regulated at late-log phase (Fig. 2).

Unfortunately, the RNA-seq data of the bacterium grown on OSX at late-log phase were not exploitable (see Additional file 1: Table S1). Nevertheless, at late-log phase, transcriptomic analysis of the bacterium grown on xylose and glucose showed that 70 out of 74 PULs predicted from genomic analysis (http://www.cazy.org/PULDB/) [19] were expressed similarly on both sugars. Three PULs (PUL 12, PUL 19 and PUL 47) were repressed on xylose relative to glucose, whereas one PUL (PUL 66) was induced. From their CAZyme composition, only PUL 12 and PUL 47 could be predicted to target fructans. Overall, these data indicate that glucose and xylose can be used indifferently as reference condition when analyzing PUL gene expression on complex polysaccharides, including xylans but excluding fructans (Additional file 1: Figure S3).

#### Proteomic analysis

**Fig. 3:**
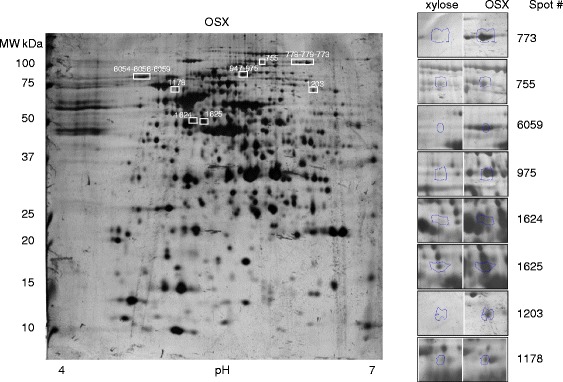
Impact of growth substrate on the soluble proteome of *B. xylanisolvens* XB1A^T^. 2-DE Analysis of the soluble proteome of strain XB1A^T^ grown on OSX versus xylose underlined the over-production of protein spots as shown on the expanded views beside the OSX 2D-gel

**Fig. 4:**
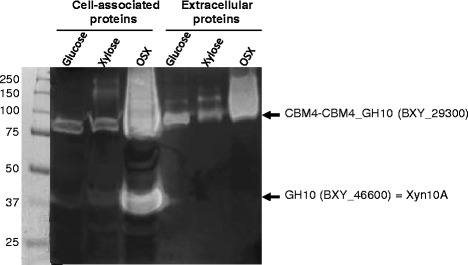
Endo-xylanase profiles of *B. xylanisolvens* XB1A^T^. Zymogram performed with OSX as substrate using cell-associated proteins (40 μg/lane) or extracellular proteins (8 μg/lane) of *B. xylanisolvens* XB1A^T^ cultivated on glucose, xylose and OSX

To investigate beyond transcription, we examined protein production of *B. xylanisolvens* XB1A^T^ cultivated on OSX relative to xylose at late-log phase, considering that the xylanase specific activity of the strain was approximately 50-fold higher on OSX (Additional file 1: Table S2). Analyzing the water-soluble protein fraction of strain XB1A^T^ on 2D-gels revealed 78 proteins that were over-produced and 57 proteins that were produced to lower levels between the two culture conditions. Among the proteins over-produced on OSX, we picked and analyzed 50 protein spots and identified eight spot regions onto the 2D-gels corresponding to 9 proteins encoded by PUL 43 (Fig. 3). Among them, the family GH10 endo-xylanase harboring two Carbohydrate-Binding Modules (CBM4) organized in tandem (BXY_29300) was the most over-produced on OSX compared with xylose (Additional file 1: Table S3). The other family GH10 endo-xylanase of 40 kDa (BXY_46600) from PUL 70, previously characterized and called Xyn10A [16] could not be visualized on the 2D-gels. Nevertheless, zymogram analysis using OSX as substrate and performed when the bacterium was grown on OSX in comparison with glucose or xylose underlined one high activity band that was only cell-associated which may be Xyn10A (BXY_46600, PUL 70), and another high activity band in both cell-associated and extracellular protein fractions corresponding to the 83 kDa GH10 protein (BXY_29300, PUL 43) (Fig. 4). One can notice that no activity band corresponding to the expected molecular mass of the other CAZymes encoded by PUL 43 *i.e. *GH5_21 (70 kDa) and CBM6-harboring GH43 (48 kDa) which have been described as endo-xylanases in other studies [21, 22] could be detected on the zymogram.

### Functional analysis of PUL 43

#### Operon organization

**Fig. 5:**
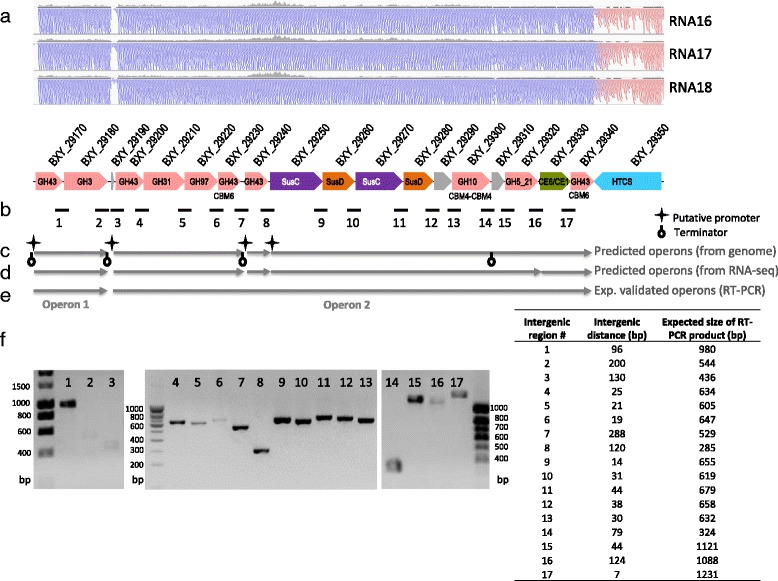
Evidence of a multi-operon organization within PUL 43. **a** Read coverage (*in grey*) within PUL 43 region and read information for that region, colored by strand, are given for the three RNA samples obtained with OSX condition. The total coverage of the genome in that region is extremely high, about 1000-4000× and was not scalable. Hence, the displayed reads were down-sampled in the window to ~50× depth. The black bars just below the grey coverage histogram for each sample indicate regions where down-sampling was performed. **b** Gene organization of PUL 43 with the position number of the neighboring gene regions used as target for the RT-PCR experiments. The intergenic distance between two genes is given in the table. **c** Operons predicted from genomic data using the FGENESB tool (see Methods). **d** Operons predicted from RNA-seq data using RockHopper (see Methods). **e** Operons experimentally validated by RT-PCR, by amplifying the intergenic regions between two consecutive genes. **f** The detection of RT-PCR fragments is shown on the agarose gel and the expected sizes of each product are given in the table

We further investigated the genetic organization of the PUL 43 cluster. This 33 kb region contains 18 ORFs (BXY_29170 to BXY_29340) that are present on the positive strand of the genome (Fig. 5). DNA sequence analysis tools (see Material & Methods) predicted the presence of three operons and of one gene being transcribed independently (Fig. 5c). This was partly validated by predictions compiling genomic and RNA-seq data (Fig. 5d). To underline the production of polycistronic transcripts, RT-PCR experiments were undertaken using RNAs extracted from strain XB1A^T^ grown on OSX. Detection of a RT-PCR product corresponding to the intergenic region #1 and the absence of amplification for the intergenic regions #2 and #3, confirmed the first operon of two genes (BXY_29170 and BXY_29180) (Fig. 5e and f). Detection of amplified fragments from intergenic regions #4 to #17 indicated that the 15 following genes (BXY_29200 to BXX_29340) are also organized in operon (Fig. 5e and f). The existence of two operons is in agreement with read coverage of PUL 43 region since no read coverage is observed in the intergenic regions #2 and #3 (between BXY_29180 and BXY_29200). In addition, the region with BXY_29240 and BXY_29250 shows much higher read coverage and contains two putative internal promoters and terminators, suggesting the existence of additional separate transcripts produced from operon 2 or post-transcriptional modifications. Our data also indicate that the two terminators predicted in operon 2 may be weak terminators resulting in a leak-through transcription of genes from the upstream promoter (5′ of BXY_29200). Collectively, these data tend to show that PUL 43 is organized in two operons, with the longest one (operon 2) possibly leading to polycistronic transcripts of different sizes, as previously observed with cellulosome-encoding operons in *Clostridium cellulolyticum* [23].

#### Mutagenesis of the sensor/regulator HTCS gene (BXY_29350)

In order to determine the importance of the xylan utilization system encoded by PUL 43, an insertion mutation into the gene encoding the HTCS (BXY_29350) was created. Indeed, HTCS mutants have proven to be useful in establishing the role of PULs in polysaccharide degradation by *Bacteroides thetaiotaomicron* [24].

**Fig. 6:**
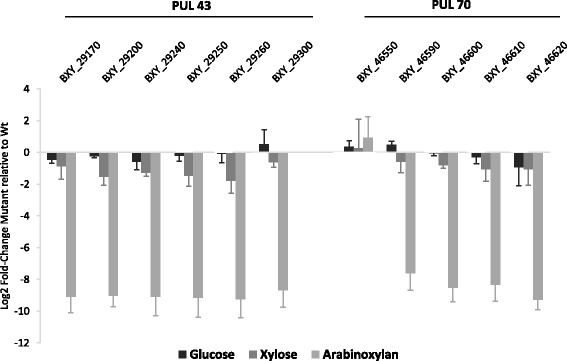
Expression measured by RT-qPCR of PUL 43 and rPUL 70 selected genes in PUL 43 *HTCS* (BXY_29350) mutant relative to *B. xylanisolvens* XB1A^T^ (Wt). Each strain was grown on WAX and harvested at mid-log phase. Each bar represents the mean of three independent experiments

The growth of the mutant was compared to that of the wild type (Wt) strain on glucose, xylose, wheat arabinoxylan (WAX) and OSX (Additional file 1: Figure S1). Both strains showed a similar growth on glucose. On xylose, the mutant growth rate and density were approximately half that displayed by the Wt strain, which did not grow as well on this sugar source than on glucose. The growth of the mutant was either strongly reduced on WAX or abolished on OSX in comparison to the Wt strain. The expression of six genes selected from the three operons in PUL 43 and five genes in PUL 70 was then analyzed in the mutant strain upon growth on WAX at mid-log phase (Fig. 6). Interestingly, four genes in PUL 70 (BXY_46590 to BXY_46620) were highly repressed in the *HTCS* mutant compared to the Wt strain, the level of the repression being similar to that of the six PUL 43 genes.

### PUL 70 (BXY_46530 to BXY_46630) microsynteny with other *Bacteroides* species

**Fig. 7:**
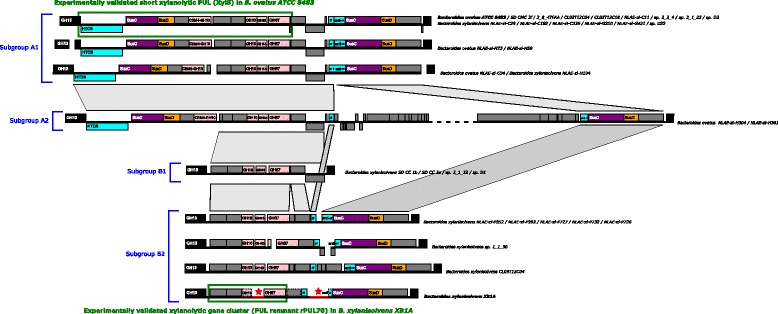
Evolution of the xylanolytic genomic region from PUL-XylS in *B. ovatus* ATCC 8483^T^ to this work on *B. xylanisolvens* XB1A^T^. Genomic regions are represented as horizontal black lines with encoded genes depicted by boxes, above or below to distinguish strands, with proportionality to intergenic distances and gene length. Genomic regions with poly-N stretches are shown in *red* and marked by a star indicating that the annotation of these regions may be incomplete. Missing gene models in *B. xylanisolvens* XB1A^T^ are depicted with *dotted boxes*. Whilst genes of unknown function are shown in *grey*, relevant gene functions are color-coded as follows: (i) glycoside hydrolase genes are represented in *pink*; (ii) PUL marker genes, *susC*- and *susD-like* genes, in *purple* and *orange*, respectively; (iii) PUL regulatory genes (HTCS, anti-σ factor/ECF-σ appear in *cyan*. The non-PUL genes, immediately flanking the PUL region of interest and conserved in all strains, are shown in *black*. Rearrangements are shown by *light-grey* polygons between conserved segments of two distinct genomic organizations. JSpeciesWS [41] was used for species assignment to either *B. ovatus* or *B. xylanisolvens* for isolates described as *Bacteroides sp.* in the JGI portal

Given the atypical transcriptional data obtained with PUL 70 genes, the synteny with other *Bacteroides*, although previously described [13], was further analyzed. Related genomic organizations were identified in the genomes of all 33 strains belonging to *B. xylanisolvens* and *B. ovatus* species, thanks to highly conserved flanking genes. All strains harbored a core of five genes, encoding three CAZymes (GH10, GH43, and GH67), one sugar transporter and an unknown protein. However, genes surrounding this core show important rearrangements. In particular, when considering the upstream region of the core, the 33 strains could be separated in two groups, as previously shown [13]: (i) group A harboring the complete xylanolytic PUL dissected in *B. ovatus* ATCC 8483^T^ (PUL-XylS or experimentally validated PUL 93 in PULDB), and (ii) group B only harboring the core of five genes which is a subset of PUL-XylS with no PUL-marker genes (*susC/D*-like or regulatory genes). Further inspection of the downstream region of this core allowed us to distinguish two subgroups for each group, depending on the presence and/or distance of a second unrelated PUL: homologous region to experimentally validated PUL 92 in *B. ovatus* ATCC 8483^T^ encoding two regulatory proteins anti-σ factor/ECF-σ, SusC/D-like proteins and one unknown protein (Fig. 7). Indeed, this downstream PUL was either two gene distant (subgroup A1), or very distant (subgroup A2) from the core in the strains with a complete PUL-XylS. In strains with only the five gene core (incomplete PUL-XylS), the downstream PUL was either absent (subgroup B1) or only separated by one gene from the core (subgroup B2). This proximity in subgroup B2 combined with the presence of poly-N stretches in *B. xylanisolvens* XB1A^T^ led to the prediction of the chimeric PUL 70, which gathered the core and the downstream unrelated PUL. In addition, the core can be considered as a remnant of PUL-XylS that is active on xylan in *B. xylanisolvens* XB1A^T^ and hence be referred to as rPUL 70.

## Discussion

The transcriptomic profiling of *B. xylanisolvens* XB1A^T^ grown on OSX versus glucose showed the over-expression of one large PUL of 21 genes (PUL 43) and of four genes of one smaller PUL of 11 genes (PUL 70). PUL 43 and PUL 70 resemble two PULs over-expressed by *Bacteroides ovatus* ATCC 8483^T^ grown on xylans that were called PUL-XylL (for large) and PUL-XylS (for small) (also respectively referred as experimentally validated PUL 73 and PUL 93) [24].

The proteomics results supported the up-regulation of several genes belonging to PUL 43 on OSX, and therefore confirmed the inducible nature of this xylan-utilization locus. They also showed the important over-production of a GH10 endo-xylanase (BXY_29300). This enzyme has homologs in several *Bacteroides/Prevotella* species [25]. One essential feature of this enzyme is that it presents a peculiar modular architecture consisting of a catalytic module disrupted by two CBM4. It has been shown that these tandemly arranged CBMs confer to the cognate GH10 enzyme a high binding capacity to xylan and it was suggested that such enzymes play a critical role in xylan degradation [25].

To further decipher the functionalities of PUL 43, a *HTCS* (BXY_29350) insertion mutant was generated and the effect of the mutation on bacterial growth and on gene expression in PUL 43 and PUL 70 was analyzed. The mutation had no effect on growth on glucose but had a substantial effect on growth on xylose. Growth reduction on xylose cannot be due to the repression of gene BXY_46610 encoding a sugar transporter (Fig. 6) but must somehow be caused by a defect in xylose utilization by the bacterium. Furthermore, the *HTCS* mutation had a dramatic effect on bacterial growth on insoluble OSX and soluble WAX. It also strongly reduced the expression of both PUL 43 and PUL 70 genes when the bacterium was grown on WAX. In PUL 43, genes belonging to the two operons were repressed, indicating that the HTCS protein regulates expression of the two operons. In PUL 70, three genes (BXY_46590, BXY_46600, BXY_46620) encoding CAZymes (GH67, GH10, GH2) and one gene (BXY_46610) encoding a sugar transporter were repressed while the gene encoding the SusC-like protein (BXY_46550) was not. This result highlights for the first time a cross-regulation between two PULs, with the HTCS regulator in PUL 43 controlling directly or indirectly the expression of genes in PUL 70.

The fact that several genes in PUL 70 *i.e.* BXY_46540, BXY_46550, BXY_46580 and BXY_46630 encoding SusC/D-like proteins, an ABC transporter and a GH13 protein, respectively were not over-expressed upon growth on OSX suggests that OSX is not the primary substrate of this PUL and/or that the boundaries of this PUL need to be redefined. Our analysis of the organization of PUL 70 and its homologs in 33 strains of *B. ovatus* and *B. xylanisolvens* highlighted four subgroups of strains harboring different genetic organization around a core cluster of five genes common to all strains. Subgroups A1 and A2 possess PUL-XylS described in *B. ovatus* [13], whereas subgroups B1 and B2 only harbor the five gene core which was not predicted as a PUL except in strain *B. xylanisolvens* XB1A^T^. We propose here that PUL 70 is a PUL chimera and the part corresponding to the five gene core was named rPUL 70 as it may be a remnant of PUL-XylS. All strains from groups B2 only assigned to *B. xylanisolvens* species might then function as *B. xylanisolvens* XB1A^T^.

Apart from PUL 43 and rPUL 70, the transcriptomics RNA-seq profiles obtained with OSX highlighted a low induction of 15 PULs relative to the glucose condition; the same PULs were also slightly up-regulated in another transcriptomic RNA-seq study using citrus pectin [20]. These cross-inductions between OSX and citrus pectin could be due to the presence of contaminating polysaccharides in both substrates. As a matter of fact, starch was detected in both substrates (0.9 % in OSX and 0.7 % in citrus pectin) and PUL 71 (BXY_47580 to BXY_47670) was induced on both substrates. It could also be due to the presence of common oligosaccharide motifs in OSX and pectin *i.e.* arabinose-containing side chains targeted by PUL 13 (BXY_15640 to BXY_15720). Finally, cross-regulations among PULs might occur as shown here between PUL 43 and rPUL 70.

From what is already known on PUL functioning in *Bacteroides* [13, 17, 18], we suggest that PUL 43 encodes proteins that bind to xylans (SusD-like proteins, CBM4-containing GH10), initiate their degradation at the surface of the bacterium (CBM4-containing GH10, maybe other CAZymes), and allow the transfer of oligosaccharides to the periplasm (SusC-like transporters), while rPUL 70 encodes enzymes (GH10, GH67, GH2) completing the degradation of oligosaccharides in the periplasm, supported by enzymes encoded by PUL 43. rPUL 70 may also be involved in the transport of the sugars from the periplasm to the cytoplasm via the sugar transporter encoded by BXY_46610.

Rogowsky et al. [13] proposed a model of xylan degradation by *B. ovatus* ATCC 8483^T^ that is different, especially because of the different CAZyme composition of PUL-XylL (or PUL 73). Indeed, this PUL would be dedicated to highly substituted glucuronoarabinoxylans (from corn, rice and sorghum) which require GH30- and GH98-encoding genes that are not present in PUL 43 of *B. xylanisolvens* XB1A^T^. According to the Rogowski model, PUL-XylS (or PUL 93) would act only on poorly decorated xylans (or simple xylans) like OSX. It is interesting to note that it is the deletion of the small PUL (PUL-XylS) and not of the large PUL (PUL-XylL) that abolished the growth of *B. ovatus* on simple xylans [13]. It occurs that PUL-XylL of *B. ovatus* encodes an inactive GH10 in the so-called “core xylan utilization cluster” while PUL-XylS harbors two genes encoding a GH10, one of them being the peculiar CBM4-containing GH10 [13]. Contrary to *B. ovatus* ATCC 8483^T^, the CBM4-containing GH10 of *B. xylanisolvens* XB1A^T^ is encoded by PUL 43 and the other GH10 is encoded by rPUL 70, and through the PUL 43 *HTCS* mutation, we showed that both genomic regions, linked at the transcriptional level, are necessary for *B. xylanisolvens* to grow on xylans (OSX and WAX). The CBM4-containing GH10 was also shown to hydrolyze xylan backbone of high degree of polymerization (DP > 5) [25] and to be located at the cell surface of *B. ovatus* ATCC 8483^T^ [13], indicating that this enzyme initiates the process of xylan breakdown. *B. intestinalis* DSM 17393^T^, *B. cellulosilyticus* DSM 14838^T^, (and *P. bryantii* B_1_4 - DSM 11371 from the bovine rumen) also harbor one gene encoding a CBM4-containing GH10 in their genomes although in PULs with different organization than those of *B. ovatus* ATCC 8483^T^ and *B. xylanisolvens* XB1A^**T**^ (http://www.cazy.org/PULDB/). Interestingly, we found that two strains of *B. xylanisolvens* (SD-CC-1b and SD-CC-2a) that did not possess that gene did not grow on WAX and had a very low growth on OSX, contrary to *B. xylanisolvens* XB1A^T^, *B. ovatus* ATCC 8483^T^, *B. intestinalis* DSM 17393^T^ and *B. cellulosilyticus* DSM 14838^T^ (not shown). From all these observations, we propose that this peculiar enzyme is crucial for xylan breakdown by human gut symbionts of the *Bacteroides* genus and that it could be considered as a functional marker of simple xylan degradation in the human gut.

## Conclusions

We have shown that the xylanolytic function of *B. xylanisolvens* XB1A^T^ involves one PUL and one PUL remnant that are linked at the transcriptomic level and that produce the necessary arsenal of CAZymes and accessory proteins for an optimal utilization of xylans of low complexity. This study, along with another recent study on *B. ovatus* ATCC 8483^T^ [13], shows that *B. xylanisolvens* XB1A^T^ belongs to a group of strains that display PUL functionalities and specificities regarding xylan utilization that are different from those displayed by other groups such as the one comprising *B. ovatus* ATCC 8483^T^ [13]. These differences may provide ecological advantages of one group of strains over the others, depending on the availability of xylans in the diet but also on their structural complexity. Hence the nature of the xylan consumed in the diet and the bacterial species selected upon that diet may lead to different oligosaccharides with different prebiotic effects. More studies are needed to better understand the function and ecology of PCW-degrading bacteria in the colon, including bacterial representatives of the Firmicutes phylum, in order to better predict the impact of diet and dietary fibers on health.

## Methods

### Bacterial strain, media, and growth conditions

*B. xylanisolvens* XB1A^T^ (DSM 18836^T^) was grown anaerobically at 37 °C in BX medium containing clarified rumen fluid [8] and 5 g/L of complex substrates (insoluble oat-spelt xylan or OSX, Serva, France; washed twice in distilled water and autoclaved to remove free sugars, and soluble wheat arabinoxylan or WAX, Megazyme, France) or sugars (glucose or xylose). The media were prepared, dispensed and inoculated by using strictly anaerobic techniques in Balch tubes. A 2.5 % (v/v) inoculum of culture pre-adapted on each substrate was used for inoculation. Bacterial growth on glucose, xylose and WAX was followed by optical density of the culture at 600 nm (OD_600nm_) recorded directly in Balch tubes using a Jenway 6320D spectrophotometer. Because OSX interfered with OD_600_ measurements, growth on this substrate was monitored by estimating the amount of bacterial proteins in the culture using the Bradford Protein Assay [26]. Three independent cultures were performed for each substrate condition i.e. glucose, xylose, WAX and OSX for subsequent transcriptomic and/or proteomic analyses (Table 1).

### OSX and WAX composition

The analysis of OSX was carried out as previously described [20] in order to determine its composition in monosaccharides, methylester groups and starch. Uronic acids were measured spectrophotometrically by the m-hydroxydiphenyl assay using galacturonic acid as external standard. The difference in response of glucuronic acid and galacturonic acid in the presence and absence of tetraborate was used to quantify them in OSX (Additional file 1: Table S4) [27]. The composition of OSX used in this study is comparable with a glucuronoxylan poorly substituted with arabinose (Xyl/GlcA/Ara 84/8/1); OSX also contains small amounts of starch (0.9 %) and possibly pectin, although pectin has never been quantified in OSX [28–30].

The WAX substrate used in this study corresponded to medium viscosity WAX (lot 40302b, Megazyme) and its composition was provided by the supplier. It is 95 % pure with a Xyl/Ara sugar ratio of 62/38, and contains 3.7 % proteins in addition to traces of starch (0.09 %) and β-glucan (0.1 %).

### Preparation of enriched fractions of mRNAs

Total RNAs were isolated from cultures harvested at mid- and late-log phase using a modified guanidinium–phenol–chloroform procedure previously described for rumen fluid [31]. Briefly, bacterial cultures (4 tubes × 8 ml) were centrifuged for 15 min at 3,000 g at 4 °C. The pellets were resuspended in 9 ml of a RNA-E solution containing solution D [32], water saturated phenol, sodium acetate 0.2 M pH 4.0 and 2-mercaptoethanol (1:1:0.1:0.007). Cells were then disrupted by bead beating for 1 min with 0.1 g zirconia beads (0.1 mm) followed by a 2-min incubation at 60 °C. These two steps were then repeated. After addition of 3.75 ml of chloroform, the samples were briefly mixed, incubated for 15 min on ice and centrifuged (12,000 g, 20 min, 10 °C). The RNAs contained in the aqueous supernatants (approximately 6 ml) were precipitated with 0.25 volume isopropanol and washed with 1 volume 75 % cold ethanol in DEPC-treated water. Total RNAs were solubilized in 100 μl of DEPC-treated water. Genomic DNA was removed using the Turbo DNA-Free DNAse (Ambion, France) for 30 min at 37 °C. RNAs were quantified using a ND-2000 NanoDrop spectrophotometer (Nanodrop Technologies, France). Enriched fractions of mRNAs were prepared using the MicrobExpress™ Bacterial mRNA Purification kit (Ambion, France). The high RNA quality and the reduction in 16S and 23S rRNA in enriched fractions of mRNAs were confirmed using an Agilent 2100 Bioanalyser (Agilent technologies, France).

### RNA-seq analyses

cDNA libraries were prepared with 100 ng of mRNA-enriched fractions following the protocols of the Illumina TruSeq Stranded Total RNA Library prep kit. The final libraries had an average fragment size of ∼ 250 bp and were quantified by qPCR before being sequenced with an Illumina HiSeq 2000 instrument on a single lane in paired end reads. The data are available in GEO datasets at NCBI (http://www.ncbi.nlm.nih.gov/) under the accession number GSE74379. Approximately 8 to 9 million paired reads per sample were obtained (Additional file 1: Table S1). Quality filtering and adapter trimming were performed with Trimmomatic v0.30 [33] using Illumina TruSeq3 adapter sequences for adapter clipping.

The *B. xylanisolvens* XB1A^**T**^ genome (GenBank accession NC_021017.1) was indexed using novoindex, and reads aligned with novoalign v. 3.00.05 (http://www.novocraft.com) against the indexed genome. For downstream gene expression analysis only the aligned R1 reads were used; these were extracted from the paired-end alignment file using samtools v0.1.19 [34] using the bitwise flag for the first read for a read pair.

Gene counts were determined for the aligned data using featureCounts v. 1.4.3-p1 [35] and the NCBI GFF3 feature file (obtained from the NCBI FTP site Dec. 2013).

Differential gene expression was performed using R 3.0.0 using edgeR/limma [36, 37]. Samples were assessed for potential outliers based on counts using normalized counts (RPKM = Reads per Kilobase per Million) that would affect downstream analyses. From this analysis we determined that samples mRNA 1, 2, 3 and 10 were problematic and thus removed them from the analysis (Additional file 1: Table S1). Counts per million (CPM) mapped reads were calculated per gene; genes with more 1 CPM in three or more samples were retained in the final edgeR analysis. Samples were normalized using edgeR’s TMM normalization. A simple generalized linear model was generated using the aforementioned filtered data from all remaining samples, and simple contrasts based on carbon source and growth stage were used to determine genes differentially expressed under the conditions shown. A separate coordinate analysis was performed using Rockhopper v. 1.30 [38] on all mRNA samples (excluding mRNA 1, 2, 3 and 10) again using data obtained from NCBI GFF3 feature file as mentioned above. Results from this analysis were primarily used to find potential groups of genes that may be expressed as a single transcriptional units or operons.

### *In silico* prediction of operons from genomic sequences

Putative promoters and terminators were searched within intergenic sequences (>100 bp) using different tools (BPROM, PPP, Arnold) available at http://molbiol-tools.ca/Promoters.htm. Operon prediction was carried out using FGENESB, which is based on distances between ORFs and frequencies of different genes neighboring each other in known bacterial genomes, as well as on promoter and terminator predictions (http://www.softberry.com/berry.phtml?topic=fgenesb&group=programs&subgroup=gfindb).

### Reverse transcription (RT) followed by PCR or quantitative PCR (qPCR)

Total RNAs (1 μg) were reverse-transcribed into cDNAs using random hexamer primers (Invitrogen, France) and 200 U SuperscriptII Rnase H^−^ reverse transcriptase (Invitrogen, France) according to the procedure supplied with the enzyme. For each RNA sample, a negative RT (no addition of reverse transcriptase) was performed and used as a negative control in subsequent PCR and qPCRs.

The presence of a polycistronic mRNA transcribed from PUL 43 of *B. xylanisolvens* XB1A^T^ was determined by performing PCR using cDNAs prepared from bacterial cultures on xylan and primer pairs designed to amplify the intergenic regions between two consecutive ORFs within the locus *i.e.* forward primers targeting the 3’ end of one ORF and reverse primers targeting the 5’ end of the following ORF (Additional file 1: Table S5). PCR was carried out with the HotMaster Taq DNA polymerase (5PRIME, Deutschland) following the instructions given for the enzyme.

The relative expression of PUL target genes in the OSX culture condition (mid and late-log phases) versus the sugar conditions was performed by quantitative PCR using a Mastercycler ep Realplex 2S (Eppendorf, France) and Quantifast SYBR Green PCR mastermix (Qiagen, France) using the supplier’s instructions. The designed specific primers are listed in the Additional file 1: Table S6. The fold change in gene expression (OSX versus glucose or xylose) was calculated from 3 biological replicates (+ two technical replicates) according to Livak and Schmittgen [39] using the 16S rRNA reference gene for normalization. Log2 fold-change at mid- and late-log phase were considered as significantly different at *p* < 0.01 (Student’s t-test).

### Preparation of bacterial soluble proteins

Bacterial soluble proteins (= cell-associated proteins) were prepared from xylose and OSX cultures (500 ml) harvested at late-log phase. Bacterial cell pellets were first washed 3 times in distilled water containing a protease inhibitor (Complete EDTA free protease inhibitor cocktail, Roche, France) and re-suspended in 1/25 volume of the same solution. Cells were broken by one passage at 2000 bars using a One Shot cell Disruptor (CellD, France). The obtained suspension was treated with 270 U endonucleases (Dnase + RNase, Sigma, France) for 30 min at ambient temperature. Unbroken cells were removed by centrifugation (23,000 g, 40 min, 4 °C). The supernatant was subsequently submitted to ultracentrifugation (100,000 g, 1 h, 4 °C) to obtain a final supernatant containing bacterial soluble proteins that were aliquoted and frozen. Before analysis, an aliquot of protein solution was mixed with 3 volumes of ice-cold acetone and kept at -20 °C for at least 2 h. After centrifugation (13,000 g, 30 min, 4 °C), the protein pellet was dried under vacuum for 5 min and solubilized in isoelectric focusing (IEF) buffer (7 M urea, 2 M thiourea, 4 % CHAPS, 0.2 % triton X100). The amount of proteins solubilised in IEF buffer was determined with the PlusOne™ 2-D Quant kit (Amersham, France) according to the supplier’s instructions.

### Two-dimensional gel electrophoresis (2-DE) and Image analysis

2-DE was performed with IPG strips of 17 cm with a linear gradient of pH 4-7 (Bio-Rad, France). They were submitted to passive (9 h) and active (9 h, 50 V) rehydration with 300 μl IEF buffer containing 0.4 % DTT, 0.15 (v/v) % Biolyte pH 4–6, 0.15 (v/v) % Biolyte pH 5–7 and 100 μg proteins. IPG strip rehydration and IEF were conducted in a focusing tray using the Bio-Rad PROTEAN® IEF System at a temperature of 20 °C. Focusing conditions were 30 min at 200 V, 1 h at 1 kV, linear voltage ramp to 10 kV for 6 h followed by a plateau at 10 kV until total 54 kV.h was reached. Focused IPG strips were stored at –20 °C in glass tubes. Prior to the second dimension, strips were equilibrated twice for 30 min in an equilibration solution (50 mM Tris–HCl pH 8.8; 6 M urea; 30 % v/v glycerol, 2 % w/v SDS) containing 130 mM DTT for the first step and 135 mM iodoacetamide and trace of bromophenol blue for the second step. The second dimension (SDS-PAGE) was carried out with 12 % polyacrylamide gels in a PROTEAN® II XL Cell (Bio-Rad, France). Proteins were visualized by silver-staining according to Bradford et al. [40].

Silver stained 2-DE gels were scanned with a GS800 imaging densitometer (Bio-Rad, France) and spot detection and image analysis were performed with the PD-Quest software (version 7.1, Bio-Rad). Three reproducible gels from each independent culture for each bacterial growth condition (OSX, xylose) were selected and included for image analysis.

Statistical image analysis was performed using Progenesis Samespots software, version 4.1 (Nonlinear Dynamics). For each independent analysis, gels were aligned on a reference gel (xylose condition). After background subtraction, spot autodetection and quantification were performed by the software. Each spot was assigned a relative value corresponding to a spot volume. To compare the OSX to the xylose condition, an experimental design was set up with these two conditions, where corresponding gels were added. Differential spot intensity was considered significant at *p* < 0.01 using ANOVA (Analysis of Variance) procedure.

### Identification of proteins by mass spectrometry

Spots of interest were excised in the 100 μg loaded silver-stained gels and subjected to the following treatments. First, the spots were unstained in a 30 mM potassium ferricyanid-100 mM sodium thiosulfate solution for 2 min and rinsed twice with MilliQ water for 15 min. They were then washed in 25 mM ammonium bicarbonate (pH 8.5)–5 % acetonitrile for 30 min and twice in 25 mM ammonium bicarbonate–50 % acetonitrile for 30 min each. The spots were then dehydrated with 100 % acetonitrile. The dried gels were reswelled in 25 mM ammonium bicarbonate containing 20 ng/μL^− 1^ trypsin. Digestion was performed at 37 °C for at least 5 h. The resulting peptides were extracted with 100 % acetonitrile. After 15 min at 37 °C, each sample was mixed with saturated cyano-4-hydroxycinnamic acid onto the matrix-assisted laser desorption ionization-time of flight (MALDI-TOF) target. Using MALDI-TOF mass spectrometry (MS) (Voyager DE-Pro, Applied BioSystems) and Voyager software for data collection and analysis, positive-ion MALDI mass spectra were recorded in the reflectron mode. Identifications by nano-liquid chromatography (LC) coupled to electrospray ionization (ESI) and tandem mass spectrometry (MS/MS) (LTQVelos, Thermo Scientific) were performed when identification from MALDI-TOF MS failed. Monoisotopic peptide masses were assigned and used for database searches with Mascot v2.2.0. Interrogations were performed against a home database containing distinct entries corresponding to the predicted mature proteins in *B. xylanisolvens* XB1A^T^. The following parameters were considered for the searches: a maximum ion mass tolerance of 25 or 50 ppm, possible modification of cysteines by carbamidomethylation, as well as partial oxidation of methionine.

### Construction of a *HTCS* mutant within PUL 43

An insertion mutation was created into the sensor/regulator *HTCS* gene (BXY_29350) of PUL 43. An internal fragment corresponding to the sensor domain of the protein (841 bp) encoded by BXY_29350 was cloned into the pGERM suicide vector (Additional file 1: Table S7 and Figure S4). The resulting construct was transformed into *Escherichia coli* WM3064 and transferred to *B. xylanisolvens* XB1A^T^ by conjugation as previously described [20]. Plasmid insertion into the target gene was then verified by PCR using primers targeting junction regions between pGERM and PUL 43 *HTCS* gene (Additional file 1: Table S7 and Figure S4).

### Ethics

Rumen fluid used to prepare growth media was collected in the experimental slaughterhouse at INRA, Saint-Genes-Champanelle, France, from animals slaughtered in accordance with the guidelines of the local Ethics Committee and current INRA ethical guidelines for animal welfare (Permit number: 63345001).

### Consent to publish

Not applicable.

### Availability of data and materials

The data sets supporting the results of this article are included within the article and its additional supplementary file. The transcriptome data are available in GEO datasets at NCBI (http://www.ncbi.nlm.nih.gov/) under the accession number GSE74379.

## Additional file

Additional file 1:
**Table S1.** RNA-seq mapping assessment. **Table S2.** Xylanase specific activity of *B xylanisolvens* XB1A^T^. **Table S3.** Proteins identified by MALDI-TOF MS or LC-ESI-MS/MS over-produced upon growth of *B. xylanisolvens* XB1A^T^ on OSX relative to xylose. **Table S4.** Composition of the commercial oat-spelt xylan (SERVA, France) used in this study. **Table S5.** Primers used for RT-PCR (to amplify the intergenic regions between two consecutive ORFs within PUL 43). **Table S6.** Primers used for relative RT-qPCR. **Table S7.** Primers used for insertion mutagenesis into PUL 43 *HTCS* gene (BXY_29350). **Figure S1.** Growth of *B. xylanisolvens* XB1A^T^ (Wt) and PUL 43 *HTCS* (BXY_29350) mutant on glucose, xylose, wheat arabinoxylan (WAX) and oat-spelt xylan (OSX). **Figure S2.** *B. xylanisolvens* XB1A^T^ gene expression in response to oat-spelt xylan (OSX) and xylose relative to glucose obtained from RNA-seq analysis. **Figure S3.** *B. xylanisolvens* XB1A^T^ PUL expression in response to xylose relative to glucose at late-log phase obtained from RNA-seq analysis. **Figure S4.** Schematic layout of the mutant construction and validation of pGERM*:HTCS* insertion into PUL 43 *HTCS* gene (BXY_29350) of *B. xylanisolvens* XB1A genome. (XLSX 454 kb)

## Abbreviations

CAZyme: carbohydrate-active enZyme
CBM: carbohydrate-binding module
HTCS: hybrid two-component system
OSX: oat-spelt xylan
PCW: plant cell wall
PUL: polysaccharide utilization locus
RPKM: reads per kilobase per million
WAX: wheat arabinoxylan
